# The Important Role of Superoxide Dismutase 2 in Controlling Poxvirus Proliferation and Pathogenicity

**DOI:** 10.3390/antiox15081019

**Published:** 2026-08-15

**Authors:** Xiaoshuang Shi, Jiamin Wang, Letian Li, Quan Liu, Jianfeng Zhang, Chang Li, Shouwen Du

**Affiliations:** 1 Institute of Basic Medical Sciences Chinese Academy of Medical Sciences, School of Basic Medicine Peking Union Medical College, Beijing 100005, China; shixs96@163.com; 2 Key Laboratory for Prevention and Control of Avian Influenza and Other Major Poultry Diseases, Ministry of Agriculture and Rural Affairs, Key Laboratory of Livestock Disease Prevention of Guangdong Province, Institute of Animal Health, Guangdong Academy of Agricultural Sciences, Guangzhou 510640, China; wangjiamin956@outlook.com (J.W.); zhangjianfeng@gdaas.cn (J.Z.); 3 State Key Laboratory of Pathogen and Biosecurity, Key Laboratory of Jilin Province for Zoonoses Prevention and Control, Academy of Military Medical Sciences, Changchun 130122, China; letian823@163.com; 4 Chinese Medicine Guangdong Laboratory, Hengqin 519031, China; liuquan1973@hotmail.com

**Keywords:** superoxide dismutase 2, poxvirus infection, ROS, antiviral role, mitochondria

## Abstract

Superoxide dismutase 2 (SOD2), a key mitochondrial antioxidant enzyme, is essential for maintaining cellular redox homeostasis by scavenging superoxide radicals. While viruses often induce oxidative stress, the specific role of SOD2 in antiviral defense remains unclear. Here, we report that vaccinia virus (VACV) infection triggers mitochondrial and cellular reactive oxygen species (ROS) and selectively upregulates SOD2, but not SOD1. Genetic knockout of SOD2 exacerbated mitochondrial ROS (mtROS) accumulation and significantly enhanced VACV replication and spread, resulting in larger viral plaques. Conversely, SOD2 overexpression constrained plaque formation and suppressed viral dissemination. Mechanistically, the antiviral function of SOD2 does not strictly rely on its enzymatic activity or mitochondrial targeting, as neither the deacetylation-mimicking mutant nor the mutant lacking the mitochondrial localization signal peptide appreciably impaired its antiviral potency. Furthermore, in a rabbit model, local overexpression of human SOD2 attenuated the poxvirus lesion formation. Our findings unveil an important yet easily overlooked role of SOD2 in antiviral defense and posit it as a promising candidate for the development of host-directed therapeutics against poxviruses.

## 1. Introduction

Viral infection is a complex biological process in which virus–host interactions mediate viral replication and propagation while also triggering intracellular signaling pathways. Accumulating evidence indicates that viral pathogens induce oxidative stress, leading to the aberrant accumulation of intracellular reactive oxygen species (ROS) [1,2]. ROS, generated primarily as by-products of aerobic metabolism, can directly damage host cellular components [3,4]. Oxidative stress can modulate redox-sensitive signaling pathways, thereby inducing cellular dysfunction or apoptosis and promoting the production of inflammatory mediators [5,6,7]. Epstein–Barr virus (EBV) infection employs this mechanism to upregulate Nox2 in B-lymphocytes, thereby activating NADPH oxidase-mediated ROS production [8]. As a classic inducer of oxidative stress, Influenza A virus (IAV) infection impairs glutathione redox homeostasis and increases ROS production in lung epithelial cells, a process in which NADPH oxidase (particularly the NOX4 isoform) plays a pivotal role [9,10]. These ROS activate the p38 and ERK1/2 MAPK pathways, thereby facilitating the nuclear export of viral ribonucleoprotein (vRNP) complexes to regulate the viral life cycle [11,12].

However, the role of poxvirus infection in regulating ROS remains controversial. Some studies have reported that poxvirus infection reduces ROS levels [13,14], whereas others indicate that the late-stage ectromelia virus (ECTV) infection causes mitochondrial injury and ROS burst, resulting in mitochondrial membrane potential loss [15]. Although the precise mechanisms by which poxviruses modulate the cellular redox system remain elusive, these findings collectively point to a complex interplay between poxviruses and host oxidative stress responses, warranting further investigation. The re-emergence of monkeypox virus (MPXV) underscores the persistent threat of emerging poxvirus infections to human health and public safety [16]. Other viruses with similar biological characteristics and pathogenicity include variola virus (VARV), cowpox virus (CPXV), and vaccinia virus (VACV). Despite being the most thoroughly studied poxvirus model, VACV still exhibits poorly defined crosstalk between its replication cycle and host oxidative homeostasis.

To maintain intracellular ROS homeostasis, eukaryotic cells employ sophisticated antioxidant defense mechanisms, including the superoxide dismutase (SOD)-mediated defense system, which includes SOD1, SOD2, and SOD3. Among them, the mitochondrial-localized SOD2 is primarily responsible for scavenging superoxide radicals (O_2_^•−^), thereby maintaining mitochondrial redox balance [17]. The loss or inhibition of SOD2 leads to increased mitochondrial ROS and an altered ROS distribution pattern, triggering a cascade of events including cytoskeletal remodeling and ferroptosis [18]. Conversely, SOD2 overexpression or hyperactivation may disrupt ROS homeostasis, as exemplified by its noncanonical peroxidase activity in advanced cancer cells [19]. During viral infection, the loss or dysfunction of SOD2 may lead to immune dysfunction, rendering the host more susceptible to viral diseases caused by H1N1 [20], HCV [21], or SARS-CoV-2 [22]. This underscores the protective role of SOD2 in mitigating the clinical outcomes of viral infections. Although SOD2 is primarily recognized for its role in mitochondrial antioxidant defense, its function in poxvirus infection and pathogenesis extends far beyond mitigating oxidative injury, yet the underlying mechanisms remain poorly characterized.

In this study, we revealed the complex interplay between SOD2 and poxvirus infection and systematically demonstrated that SOD2 exerts an antiviral defense function by restricting poxviral replication and spread, which extends beyond its antioxidant role. Importantly, we provided the first evidence that prophylactic SOD2 intervention significantly mitigates poxvirus-induced skin pathology under the tested conditions. Our findings highlight its potential as a novel candidate for developing antiviral agents against poxviral infections, including monkeypox.

## 2. Materials and Methods

### 2.1. Cells and Viruses

HEK293T cells were maintained in Dulbecco’s Modified Eagle Medium (DMEM; Gibco, Carlsbad, CA, USA) supplemented with 10% fetal bovine serum (FBS) and 1% penicillin-streptomycin. A549 cells were cultured in Dulbecco’s Modified Eagle Medium/Nutrient Mixture F-12 (DMEM/F12; Gibco, Carlsbad, CA, USA) containing 10% FBS and 1% penicillin-streptomycin. Vero cells were grown in Modified Eagle Medium (MEM; Gibco, Carlsbad, CA, USA) with 10% FBS and 1% penicillin-streptomycin. All cell lines were incubated at 37 °C in a 5% CO_2_ atmosphere and were kindly provided by the Stem Cell Bank, Chinese Academy of Sciences. The VACV Tiantan strain used in this study was maintained in our laboratory. A recombinant vaccinia virus expressing mCherry (VACV-mCherry) was constructed by Dr. Du via homologous recombination and plaque purification based on the VACV Tiantan strain. The mCherry cassette was inserted downstream of the viral TK gene, driven by a VACV early/late promoter, and did not disrupt the expression of other viral genes, as previously reported [23].

### 2.2. Antibodies

Antibodies were used in this study: SOD1 monoclonal antibody (Proteintech, #67480-1-Ig, Wuhan China); SOD2 polyclonal antibody (Proteintech, #24127-1-AP, Wuhan, China); β-actin polyclonal antibody (Proteintech, #20536-1-AP, Wuhan, China); D8L polyclonal antibody (Cusabio, #CSB-PA322653LA01VAA, Wuhan, China); Acetyl-SOD2/MnSOD (K68) Antibody (Affinity Biosciences, #AF3751, Changzhou, China); Acetyl-SOD2/MnSOD (K122) Antibody (Affinity Biosciences, #AF4360, Changzhou, China); Anti-CRISPR-Cas9 antibody (Abcam, #Ab210571, Cambridge, UK).

### 2.3. Design and Construction of SOD2-Targeting Small Guide RNA (sgRNA)

The coding sequence (CDS) of the SOD2 gene (Gene ID: 6648) was retrieved from the NCBI database to identify exon regions. sgRNA sequences targeting exonic regions of the SOD2 gene were designed using the Broad Institute GPP Web Portal (https://portals.broadinstitute.org/gpp/public/, accessed on 8 July 2023), as listed in Table 1. All sgRNA sequences were commercially synthesized. For the sgRNA cloning, A phosphorylation and annealing reaction was prepared containing 1 μL each of forward and reverse primers (10 μM), 1 μL of 10× T4 ligase buffer (with ATP), 0.5 μL of T4 polynucleotide kinase (T4 PNK), and nuclease-free water to a final volume of 10 μL. The reaction was incubated at 37 °C for 30 min, heated to 95 °C for 5 min, and then cooled to 25 °C at a rate of 5 °C per minute. The annealed product was diluted 200-fold for subsequent use. The LentiCRISPRv2 vector was digested with *Bsm*B I restriction enzyme at 37 °C for 30 min, followed by gel purification of the linearized product. A ligation reaction was assembled with 50 ng of linearized vector, 1 μL of diluted sgRNA annealing product, 5 μL of 2× quick ligase buffer, nuclease-free water to 9 μL, and 1 μL of quick ligase. The mixture was incubated at room temperature for 10 min. The ligation product was transformed into Stbl3 competent cells, which were plated on ampicillin-containing solid medium. After 12 h of growth, individual colonies were selected for PCR verification. Plasmid DNA was extracted from positive clones to obtain the recombinant plasmid LentiCRISPR-sgSOD2, which was subsequently sequenced by Tsingke Biotechnology Co., Ltd. (Beijing, China) to confirm the correct sgRNA sequence.

### 2.4. Generation of SOD2 Constructs and Mutants

Using the SOD2 coding sequence from NCBI GenBank (Gene ID: NM_000636.4) as reference, we designed and synthesized oligonucleotides for full-length gene amplification. The full-length SOD2 and a truncated variant lacking the mitochondrial targeting signal (∆TP) were amplified by PCR from HEK293T cDNA and inserted into pCMV-3×Flag or pEGFP-N3 through homologous recombination. To generate the V16A, H50A, K68R, and K122R mutants, we employed the Hieff Mut^®^ site-directed mutagenesis kit (YEASEN, #11003ES10, Shanghai, China) with corresponding primers, strictly following the recommended protocol. All primers used are summarized in Table 1, and the identity of each construct was confirmed by Sanger sequencing.

### 2.5. Lentiviral Packaging and Stable Cell Line Selection

HEK293T cells were seeded in 6-well plates at a density of 2 × 10^5^ cells/mL in DMEM supplemented with 10% fetal bovine serum and cultured overnight at 37 °C in a 5% CO_2_ incubator. At 70–80% confluence, the cells were co-transfected with LentiCRISPR-sgSOD2 or LentiCRISPRv2-sgScram together with the packaging plasmids pMD2.G and pCMV-dR8.2 dvpr. After 6 h of incubation, the medium was replaced with fresh complete medium, and the cells were cultured for an additional 48 h. The lentivirus-containing supernatant was collected and filtered through a 0.45 µm filter. A549 cells were then infected with 2 mL of the filtered lentiviral supernatant containing 8 µg/mL polybrene (YEASEN, #40804ES76, Shanghai, China). Following 3–6 h of incubation at 37 °C, the viral supernatant was replaced with complete medium, and the infection was allowed to proceed for 48 h. The medium was subsequently replaced with fresh culture medium containing 2 µg/mL puromycin (Thermo Fisher Scientific, #A1113803, Waltham, MA, USA) to select for stably transduced cells.

### 2.6. Genotypic Validation of Clonal Cell Lines

Genomic DNA was extracted from monoclonal cell lines, and the target gene region was amplified by PCR under the following conditions: initial denaturation at 94 °C for 2 min; 35 cycles of denaturation at 94 °C for 30 s, annealing at 55 °C for 30 s, and extension at 72 °C for 1 min; followed by a final extension at 72 °C for 5 min. The PCR products were annealed and then digested with 1 μL of T7 Endonuclease I (T7EI; Beyotime Biotechnology, #D7080L, Shanghai, China) at 37 °C for 30 min. The digestion reaction was terminated by heating at 85 °C for 15 min. Digested products were separated by agarose gel electrophoresis, imaged, and analyzed. The purified PCR products were cloned into the pMD19-T vector, transformed into DH5α competent cells, and plated for selection. Positive clones were picked for culture, and plasmid DNA was extracted and submitted to Tsingke Biotechnology Co., Ltd. (Beijing, China) for sequencing.

### 2.7. Western Blotting

Cells were harvested and lysed in Western and IP lysis buffer (Beyotime, #P0013, Shanghai, China). Protein concentrations were determined using a BCA assay (Beyotime, #P0010, Shanghai, China), and equal amounts of protein were denatured in 5× SDS loading buffer at 100 °C for 10 min. The denatured samples were resolved by 10% SDS-PAGE and transferred onto nitrocellulose membranes (Bio-Rad, #1620112, Hercules, CA, USA). Membranes were blocked with 5% skim milk in TBST for 1 h at room temperature (RT), followed by incubation with primary antibodies for 2 h at RT. After three 10 min washes with TBST, the membranes were incubated with horseradish peroxidase (HRP)-conjugated goat anti-rabbit secondary antibody for 30 min at RT. Following three additional washes with TBST, immunoreactive bands were visualized using an enhanced chemiluminescence (ECL) substrate (YEASEN, #36208ES60, Shanghai, China) and imaged. Band intensities were quantified using ImageJ software (version 1.51j8).

### 2.8. Cell Activity Assay

A549-SOD2 KO and sgScram cells were seeded in 96-well plates at a density of 10,000 cells per well, with six replicate wells per experimental group. Cell viability was assessed at 24, 36, 48, and 72 h after plating using the Cell counting kit-8 (CCK-8; Solarbio, #CA1210, Beijing, China). Following removal of the culture medium, 100 µL of 10% CCK-8 solution in fresh medium was added to each well, and the plates were incubated at 37 °C for 1 h protected from light. Absorbance was measured at 450 nm using a Spark multimode microplate reader (Tecan, Männedorf, Switzerland) to evaluate the effect of SOD2 knockout on cell proliferation.

### 2.9. Reactive Oxygen Species (ROS)

Intracellular ROS levels were detected using 5,6-chloromethyl-2′,7′-dichlorodihydrofluorescein diacetate (CM-H_2_DCFDA; Thermo Fisher, #C6827, Waltham, MA, USA). Cells were seeded in 6-well plates and infected with vaccinia virus (VACV) after reaching adherence. At 24 h post-infection, cells were washed with phosphate-buffered saline (PBS) and incubated with 10 μM CM-H_2_DCFDA for 30 min at 37 °C in the dark. Following incubation, cells were washed three times with PBS and immediately visualized using a fluorescence microscope (EVOS FL Auto, Thermo Fisher Scientific, Carlsbad, CA, USA) for image acquisition and analysis.

### 2.10. Mitochondrial ROS (mtROS) Measurement

Cells were washed with PBS and incubated with 10 μM MitoSOX red reagent (MCE, #HY-D1055, Monmouth Junction, NJ, USA) for 30 min at 37 °C in the dark. After incubation, cells were washed three times with PBS and immediately imaged using a fluorescence microscope (EVOS FL Auto, Thermo Fisher Scientific, Carlsbad, CA, USA).

### 2.11. SOD2 Activity Assay

The SOD2 activity assay was performed using the Cu/Zn-SOD and Mn-SOD assay kit with WST-8 (Beyotime, #S0103, Shanghai, China) according to the manufacturer’s instructions. Cells were preincubated with inhibitor A for 1 h at 37 °C, followed by incubation with inhibitor B for 15 min at the same temperature. Subsequently, the samples were transferred to a 96-well plate and mixed with the assay solution. Absorbance was measured at 450 nm using a Spark multimode microplate reader (Tecan, Männedorf, Switzerland). SOD2 activity units are calculated based on the percentage inhibition rate. For detailed experimental procedures and calculation methods, refer to the operating manual for this kit.

### 2.12. Plaque Assay

A549-SOD2 KO and sgScram control cells were seeded in 12-well plates at a density of 2 × 10^5^ cells/mL. After attachment, the cells were infected with the virus inoculum for 2 h at 37 °C. The inoculum was then removed and replaced with maintenance medium (DMEM/F12 containing 2% FBS) supplemented with 1% methylcellulose to restrict viral spread. Cultures were incubated for 24 h, with cell status monitored every 12 h; the assay was terminated when distinct plaques became visible. Cells were fixed with 4% paraformaldehyde for 15 min, washed twice with PBS, and stained with 300 μL of crystal violet solution per well for 30 min at room temperature. After rinsing with distilled water, plaques were visualized and photographed. In parallel, plaque formation was also monitored by fluorescence microscopy throughout the incubation period.

### 2.13. Transmission Electron Microscopy

A549-SOD2 KO and sgScram cells were seeded into 6-well plates at 4 × 10^5^ cells/well and cultured overnight. After viral infection at 0.5 PFU/cell for 2 h, the medium was replaced with DMEM/F12 containing 2% FBS and incubated for 24 h. Cells were rinsed with PBS, harvested by scraping, and pelleted by centrifugation. The pellet was fixed with 2.5% glutaraldehyde overnight at 4 °C. Subsequent embedding, sectioning, and imaging were performed by a commercial service provider.

### 2.14. Subcellular Localization Analysis

A549 cells were co-transfected with plasmids encoding C-terminally EGFP-tagged SOD2 (or its mutants) and the mitochondrial marker COX8B-mCherry. At 48 h post-transfection, cells were fixed with 4% paraformaldehyde for 15 min, permeabilized with 0.1% Triton X-100 for 10 min at room temperature, and stained with DAPI for 10 min in the dark. After two washes with PBS, images were acquired using a Zeiss LSM 900 confocal microscope with a 63× oil-immersion objective. Co-localization of SOD2-EGFP with the mitochondrial marker was analyzed to determine subcellular distribution.

### 2.15. Animal Experiments

New Zealand white rabbits (2.5–3.0 kg) were obtained from the Guangdong Medical Laboratory Animal Center. Following dorsal hair removal, the rabbits received intradermal injections of liposome-pSOD2 complexes (30 μg per site). Two days later, the same sites were inoculated intradermally with 100 μL of VACV suspension containing 1 × 10^6^ PFU. The formation and progression of skin lesions on the backs of the rabbits were monitored at multiple time points post-infection. All animal experiments were approved by the experimental animal administration and ethics committee of the Institute of Animal Health, Guangdong Academy of Agricultural Sciences (approval No. YC-SPF2025035), and were conducted in accordance with the committee’s guidelines.

Four specific-pathogen-free female New Zealand white rabbits (2.5–3.0 kg) were obtained from the Guangdong Medical Laboratory Animal Center and randomly assigned to two groups: virus-challenged (n = 2) and non-challenged control (n = 2). Within each group, one rabbit received an intradermal injection of liposome-formulated pSOD2 (30 μg per site), and the other received the control plasmid (30 μg per site). Two days after plasmid administration, rabbits in the challenged group were inoculated intradermally at the same dorsal sites with 100 μL of vaccinia virus suspension (1 × 10^6^ PFU), whereas those in the control group received an equal volume of sterile PBS as a mock challenge. Skin lesion development was monitored at multiple time points post-infection. At the end of the experiment, all animals were euthanized by intravenous injection of sodium pentobarbital (100 mg/kg) according to an approved institutional protocol.

### 2.16. Tissue Harvesting and Protein Extraction

At the end of the experiment, rabbits were euthanized by intravenous injection of an overdose of sodium pentobarbital (100 mg/kg). Full-thickness skin biopsies (~1 cm^2^) were excised from the injection sites, immediately snap-frozen in liquid nitrogen, and stored at –80 °C. Tissue samples were homogenized in ice-cold RIPA lysis buffer supplemented with a protease inhibitor cocktail using a bead-based homogenizer. Protein concentrations were determined by the BCA assay, and SOD2 expression was analyzed by Western blotting.

### 2.17. Enzyme-Linked Immunosorbent Assay

Recombinant human SOD2 protein and heat-inactivated VACV (60 °C for 30 min) were used as coating antigens. SOD2 was diluted in coating buffer to 0.1 μg/mL, and VACV was diluted 100-fold to a final concentration of 2.5 × 10^4^ PFU/mL. Microtiter plates were coated with 100 μL of each antigen working solution per well overnight at 4 °C. After three washes with PBST, wells were blocked with 200 μL of blocking buffer (5% BSA) for 1–2 h at 37 °C. Following removal of the blocking solution and a single wash, 100 μL of rabbit serum (1:100 dilution) was added and incubated for 1–2 h at 37 °C. The wells were washed three times (5 min each), then incubated with 100 μL of HRP-conjugated goat anti-rabbit IgG secondary antibody (1:250 dilution) for 1 h at 37 °C. After three further washes, 100 μL of TMB substrate was added and incubated for 20 min at room temperature in the dark. The reaction was stopped with 50 μL of stop solution, and the absorbance was read at 450 nm and 620 nm. The calibrated value was calculated as OD_450_ vs. OD_620_. Blank and negative control wells were included in each assay.

### 2.18. Statistical Analysis

Data are expressed as mean ± SEM from three or six replicates and are representative of three independent experiments. Statistical analyses were conducted using GraphPad Prism version 10.4.1. Comparisons between groups were performed by one-way ANOVA with Dunnett’s multiple comparison test. Significance levels are indicated as follows: ns, not significant; * *p* < 0.05; ** *p* < 0.01; *** *p* < 0.001; **** *p* < 0.0001.

## 3. Results

### 3.1. VACV Infection Induces Oxidative Stress and SOD2 Expression In Vitro

**Figure 1:**
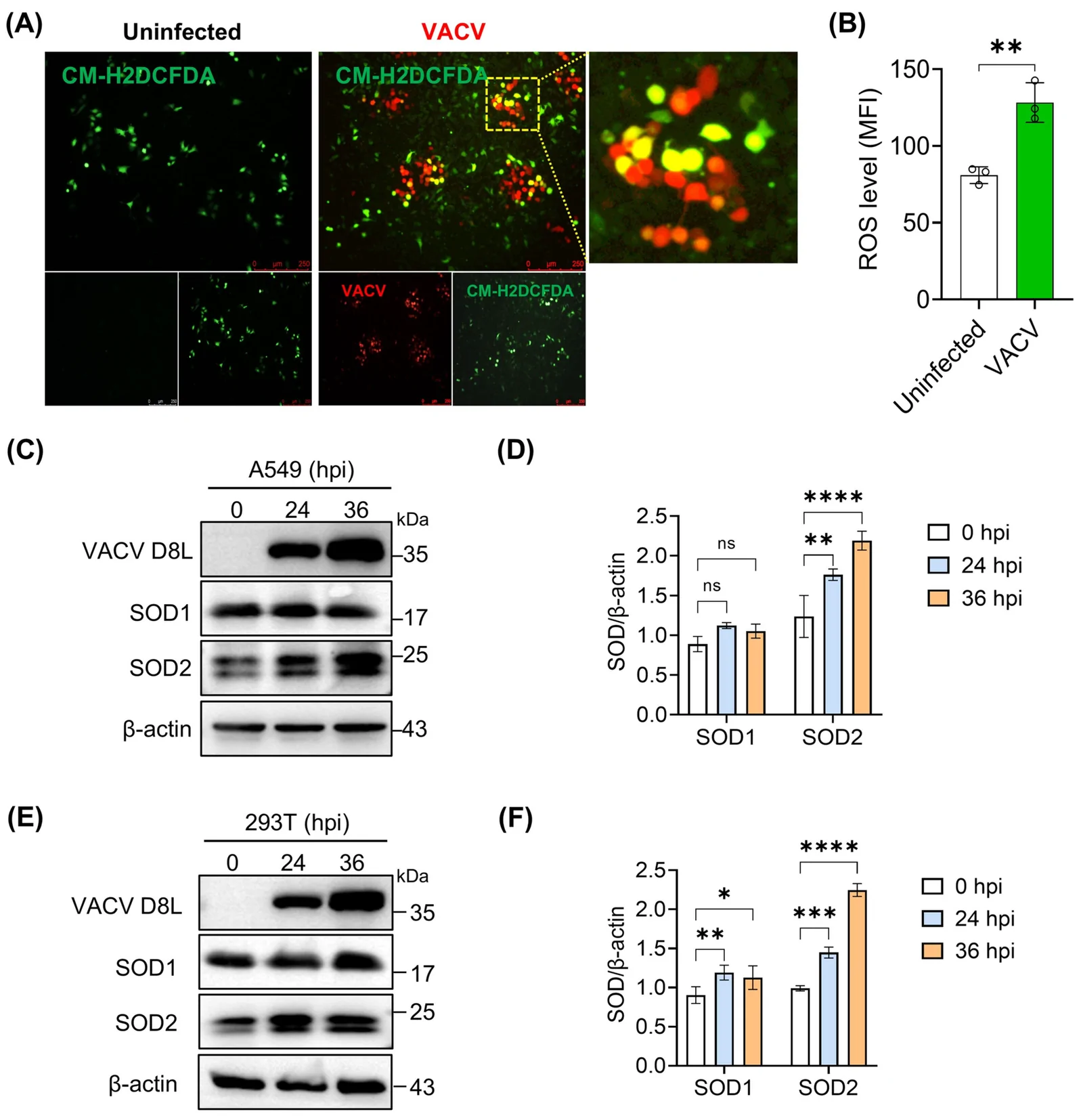
Viral infection induces oxidative stress and SOD2 expression. (**A**) Representative fluorescence images showing ROS levels in VACV-mCherry-infected cells. A549 cells were infected with VACV-mCherry (MOI = 0.1 PFU/cell), stained with CM-H2DCFDA at 24 hpi, and imaged by fluorescence microscopy. (**B**) Quantitative analysis of ROS fluorescence intensity from panel (**A**). (**C**) Western blotting analysis of SOD1 or SOD2 expression in A549 cells infected with VACV (MOI = 0.5 PFU/cell) for 24 or 36 h. β-actin served as a loading control. (**D**) Quantification of SOD1 and SOD2 levels (**C**) in the infected A549 cells. Protein levels were normalized to β-actin and are presented relative to control. (**E**) Western blotting analysis of SOD1 or SOD2 expression in 293T cells infected with VACV (MOI = 0.5 PFU/cell) for 24 or 36 h. β-actin served as a loading control. (**F**) Quantification of SOD1 and SOD2 expression (**E**) in VACV-infected 293T cells. Protein levels were normalized to β-actin and are presented relative to control. Data represent mean ± SEM (n = 3 independent experiments); * *p* < 0.05; ** *p* < 0.01; *** *p* < 0.001; **** *p* < 0.0001; ns indicates not significant.

Viral infection typically induces oxidative stress in host cells [1]. To evaluate whether poxvirus infection perturbs cellular redox homeostasis, we infected A549 cells with VACV-mCherry and labeled them with CM-H_2_DCFDA at 24 h post-infection. Fluorescence microscopy revealed a modest increase in CM-H2DCFDA signal (green) in VACV-infected cells (red), although the signal intensity varied among individual infected cells (Figure 1A). These data indicate that VACV infection globally elevates intracellular ROS levels (Figure 1B), consistent with the induction of oxidative stress reported previously [15]. Given the dual role of SODs in both mitigating ROS-induced damage and mediating ROS-dependent signaling, we next examined the expression dynamics of SOD1 and SOD2 in infected cells. We qualitatively and quantitatively analyzed the levels of VACV D8L, SOD1, and SOD2 in infected A549 and HEK293T cells at 24 and 36 hpi. VACV infection significantly upregulated SOD2 expression in a time-dependent manner, whereas SOD1 levels remained largely unchanged in A549 cells (Figure 1C–F). Taken together, these findings demonstrate that VACV infection triggers oxidative stress and induces SOD2 expression, suggesting a potential role for SOD2 in regulating VACV infection and mitochondrial redox responses.

### 3.2. SOD2 Controls the Cellular and Mitochondrial ROS Levels

**Figure 2:**
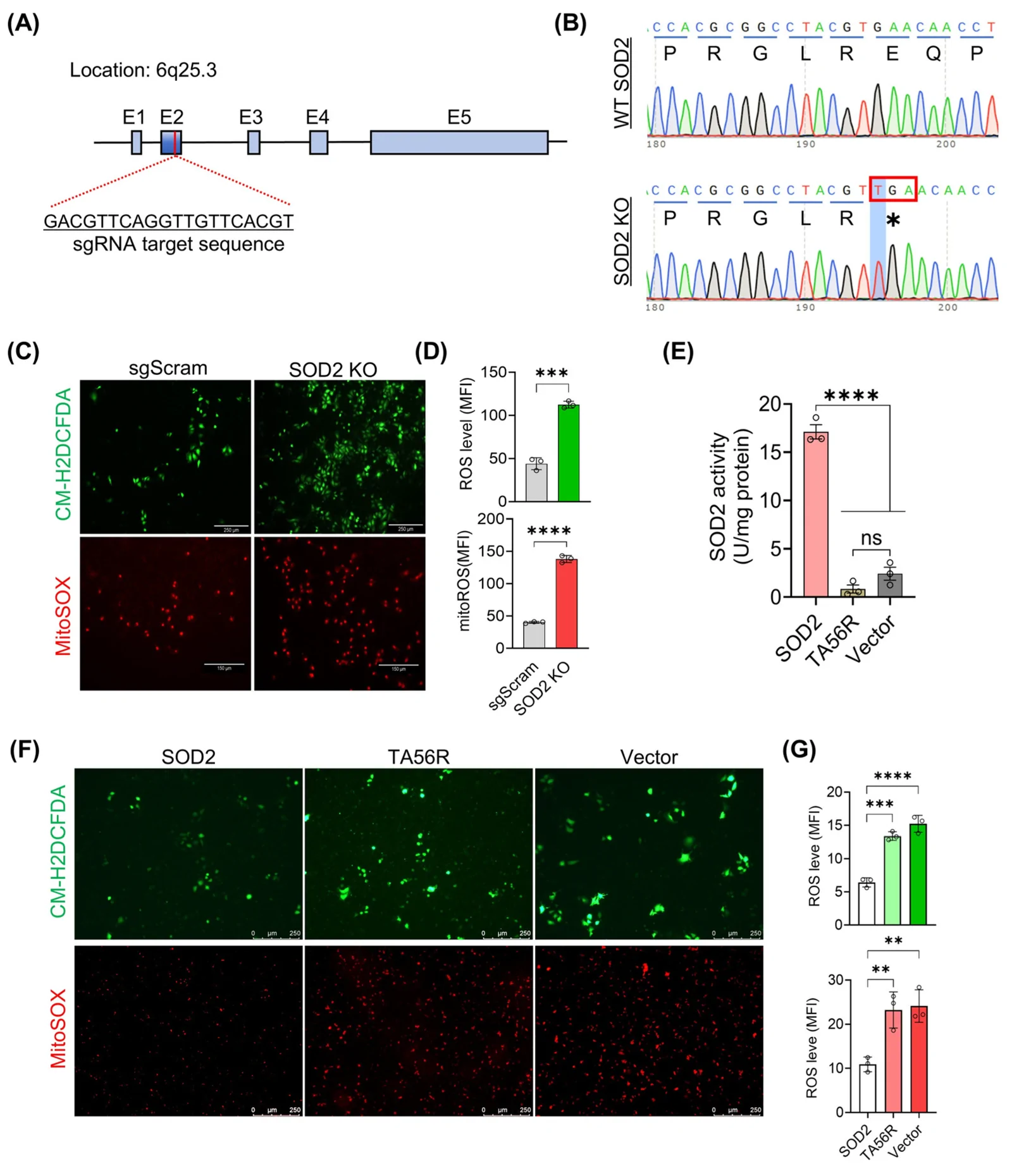
SOD2 controls the cellular and mitochondrial ROS levels. (**A**) Schematic showing the SOD2 genomic locus and the sgRNA targeting site within exon 2. (**B**) Sanger sequencing chromatograms validating the engineered mutation in A549 SOD2 KO cells. The asterisk (*) indicates the stop codon (TGA) (**C**) Representative images showing ROS levels in A549 SOD2 KO and sgScram cells. Intracellular and mitochondrial ROS levels were assessed by staining with CM-H_2_DCFDA (green, scale bar: 250 μm) and MitoSOX Red (red, scale bar: 150 μm), respectively. (**D**) Quantitative analysis of ROS fluorescence intensity from panel (**C**). (**E**) Intracellular SOD2 enzyme activity levels in SOD2 and TA56R. (**F**) Representative images showing intracellular ROS (green, scale bar: 250 μm) and mitochondrial ROS (red, scale bar: 250 μm) levels in cells expressing SOD2, TA56R, and the vector control. (**G**) Quantitative analysis of ROS fluorescence intensity from panel (**F**). Data represent mean ± SEM (n = 3 independent experiments); ** *p* < 0.01; *** *p* < 0.001; **** *p* < 0.0001. ns indicates not significant.

To further investigate the role of SOD2 in VACV infection and ROS homeostasis, we established an SOD2-deficient A549 stable cell line using CRISPR/Cas9-mediated gene editing. The specific small guide RNAs (sgRNAs) targeting exon 2 of SOD2 (chromosome 6q25.3) were designed using the Broad Institute GPP Web Portal (https://portals.broadinstitute.org/gpp/public/, accessed on 8 July 2023) and cloned into the LentiCRISPRv2 vector under the U6 promoter, yielding the recombinant plasmid LentiCRISPR-sgSOD2 (Figure 2A).

We further established a stable SOD2-knockout (KO) A549 cell line via lentiviral transduction of a SOD2-targeting sgRNA followed by antibiotic selection (Figure S1A). PCR amplification of the targeted genomic locus, followed by T7 endonuclease I (T7EI) digestion, revealed the presence of heteroduplex DNA, indicative of induced insertion–deletion mutations (Figure S1B). Sanger sequencing of the edited region confirmed a single thymine (T) insertion in exon 2 of the SOD2 gene in KO cells relative to wild-type controls, which introduces a frameshift mutation and a premature stop codon, thereby leading to truncation of SOD2 (Figure 2B). This result was corroborated by Western blot analysis (Figure S1C), confirming effective suppression of SOD2 protein expression.

Cell viability assays showed that SOD2 ablation did not appreciably affect cellular proliferation over the observation period (Figure S1D), but it significantly perturbed intracellular and mitochondrial reactive oxygen species (ROS) homeostasis (Figure 2C,D), consistent with a previous report [24] and further substantiating the critical role of SOD2 in maintaining redox balance. Given that poxviruses commonly encode a superoxide dismutase-like protein (for instance, VACV Tiantan strain harbors TA56R, a Cu-Zn superoxide dismutase-like protein, Figure S2), we next investigated whether such proteins possess intrinsic superoxide dismutase activity within the cellular context. SOD2 activity assays ruled out this possibility. The ectopic expression of TA56R alone neither exhibited SOD2 activity nor appreciably influenced intracellular ROS levels (Figure 2E–G), consistent with a previous report [25]. These observations may explain the marked ROS alterations seen during vaccinia virus infection, although the biological significance of TA56R remains to be further elucidated.

### 3.3. SOD2 Deficiency Markedly Increases Cellular Susceptibility to VACV Infection

**Figure 3:**
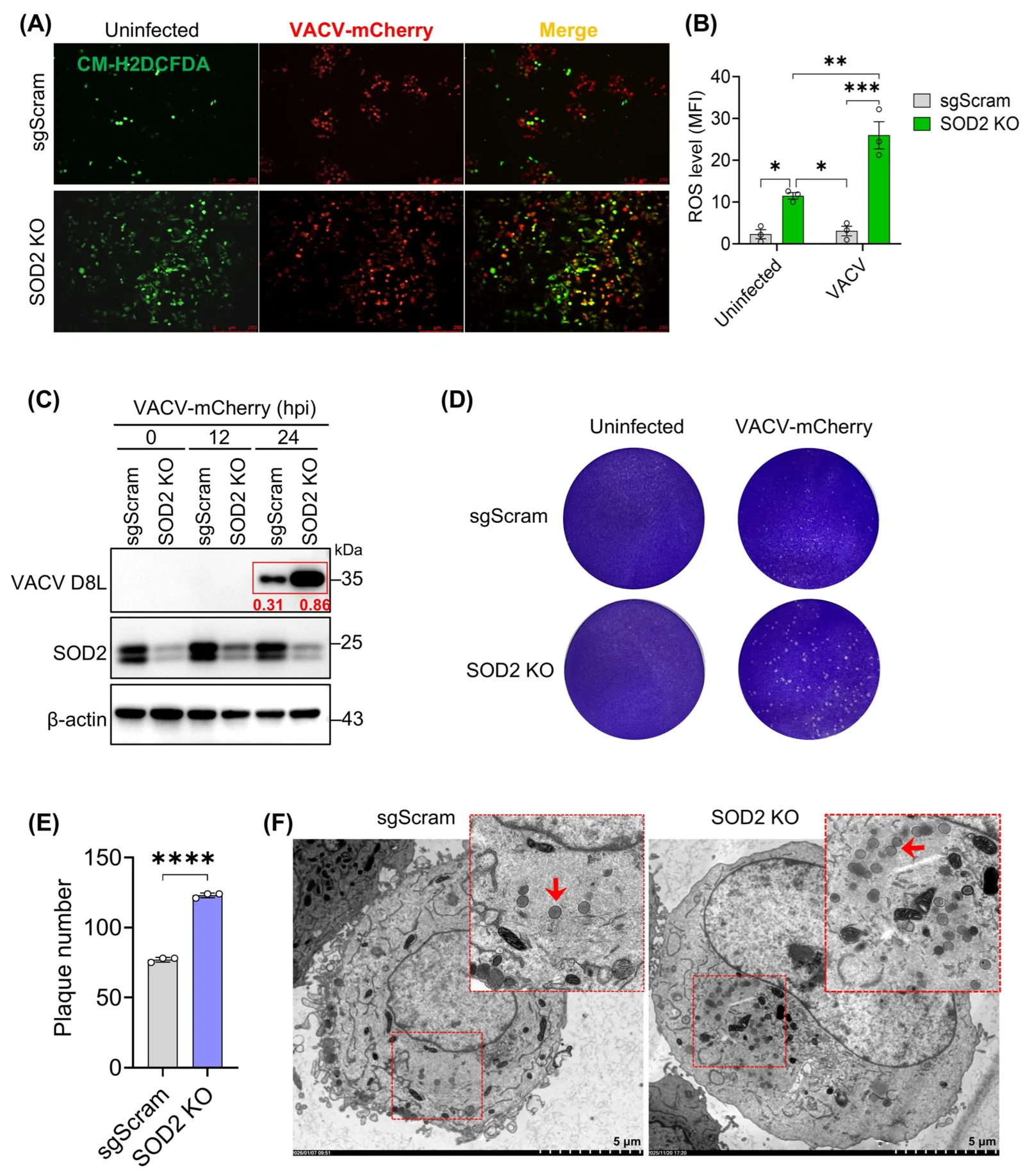
SOD2 silencing enhances vaccinia virus infection. (**A**) Representative fluorescence images of intracellular ROS in the infected A549 SOD2 KO and sgScram cells. Cells were infected with VACV-mCherry (red) at 0.5 PFU/cell and stained with CM-H_2_DCFDA (green) at 24 hpi. Scale bar: 250 μm. (**B**) Quantitative analysis of ROS fluorescence intensity from panel (**A**). (**C**) Western blotting analysis of viral D8L protein expression in VACV-mCherry-infected A549 SOD2 KO and sgScram cells. Protein levels were normalized to β-actin and are presented relative to control. (**D**) Representative images of crystal violet-stained plaques in the infected A549 SOD2 KO and sgScram cells. Cells were infected with VACV-mCherry at an MOI of 0.5 for 24 h and fixed with 4% paraformaldehyde. (**E**) Quantitative analysis of plaque formation shown in panel (**D**). (**F**) Transmission electron microscopy images showing immature viral particles in A549 SOD2 KO and sgScram cells infected with VACV (0.5 MOI) at 24 h post-VACV infection. Arrows and red areas indicate immature virus particles. Data represent mean ± SEM (n = 3 independent experiments); * *p* < 0.05; ** *p* < 0.01; *** *p* < 0.001; **** *p* < 0.0001.

Next, to assess viral fitness under high-ROS conditions resulting from SOD2 loss, SOD2 KO and sgScram cells were infected with VACV-mCherry at an MOI of 0.1 PFU/cell for 24 h. In VACV-infected SOD2-KO A549 cells, we observed a significant increase in both CM-H2DCFDA-positive (green) and mCherry-positive (red) cells (Figure 3A). Quantitative ROS analysis revealed that VACV infection further elevated intracellular ROS levels (Figure 3B), consistent with observations in normal cells (Figure 1A) and a previous study [26]. Additionally, the intracellular viral proteins analysis showed that, compared with the corresponding protein levels in sgScram cells, the VACV D8L protein levels in SOD2 KO cells were significantly higher (Figure 3C), indicating that the absence of SOD2 enhances viral protein expression and suggesting that loss of SOD2 enhances viral infectivity and fitness. Viral plaque assay confirmed this finding and showed that significantly more plaques formed in SOD2-deficient cells compared to that in the infected sgScram cells (Figure 3D,E). Transmission electron microscopy results further showed that, compared with the immature virus particles in sgScram cells, more immature particles were observed in SOD2 KO cells, and they were more concentrated (Figure 3F), which perfectly confirmed the results of the above experiments, suggesting that the endogenous SOD2 is critical for cellular defense against VACV infection.

### 3.4. SOD2 Deficiency Promotes Viral Transmission and Plaque Expansion

**Figure 4:**
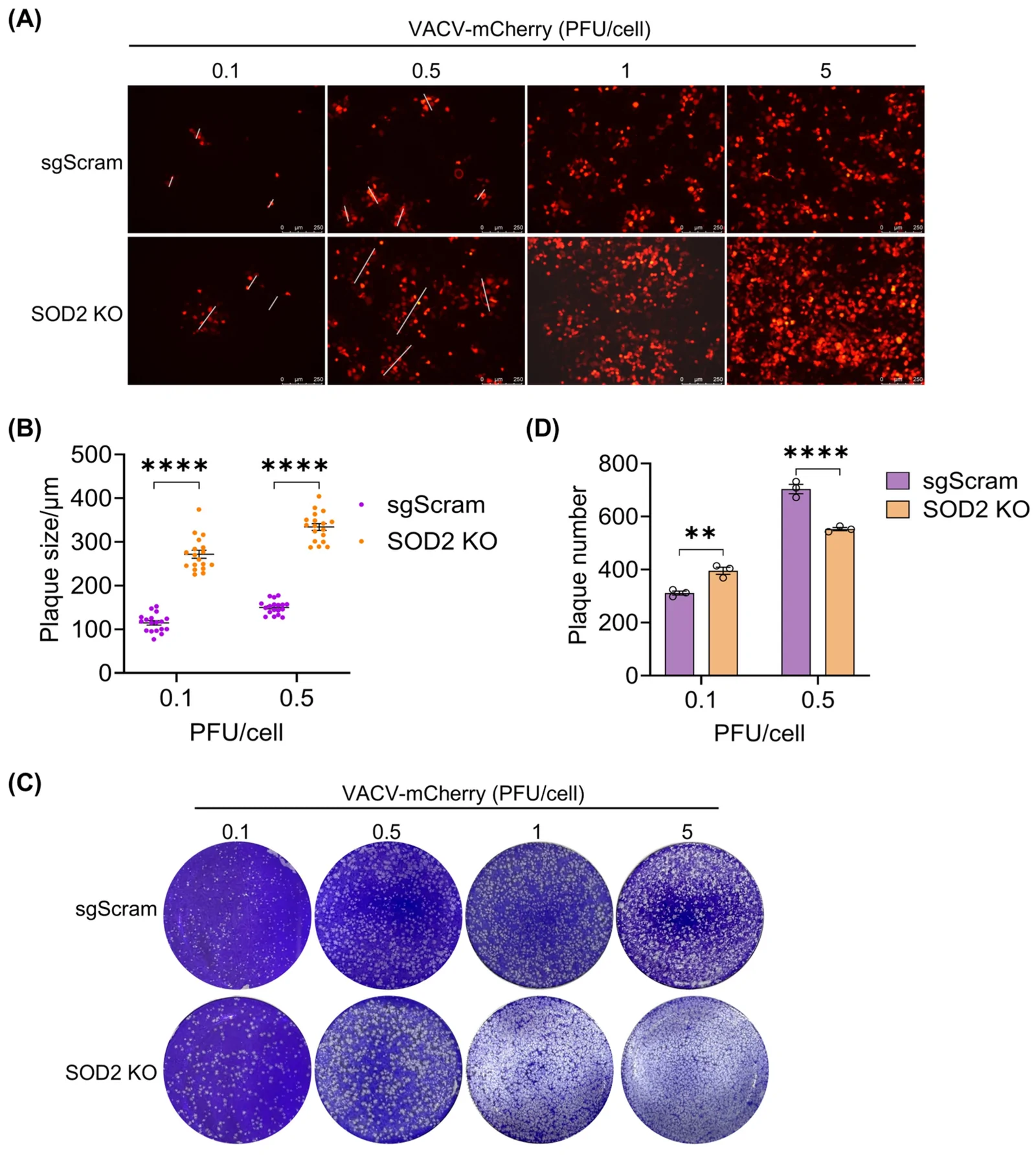
Epigenetic silencing of SOD2 accelerates viral spread. (**A**) Representative fluorescence images showing viral plaque formation in the infected A549 SOD2 KO and sgScram cells. VACV-mCherry-infected cells at indicated MOIs were maintained under 0.5% methylcellulose overlay and imaged at 24 hpi. (**B**) Quantitative analysis of plaque sizes in panel (**A**): diameters were measured and statistically evaluated. Data represent mean ± SEM (n = 18 independent experiments). (**C**) Representative images of crystal violet-stained plaques in the infected A549 SOD2 KO and sgScram cells. Cells were infected with VACV-mCherry at an MOI of 0.5 for 24 h and fixed with 4% paraformaldehyde. (**D**) Quantitative analysis of plaque formation shown in panel (**C**). Data represent mean ± SEM (n = 3 independent experiments except for the measurement of plaque size); ** *p* < 0.01; **** *p* < 0.0001.

To further investigate the role of SOD2 on viral spread, A549-SOD2 KO and sgScram cells were infected with VACV-mCherry at increasing MOIs. Fluorescent plaque analysis at 24 hpi revealed that SOD2 deficiency consistently enhanced viral dissemination (Figure 4A). All MOIs tested, viral plaques formed in SOD2 KO cells were larger than those observed in sgScram control cells. At low MOI (0.1 or 0.5), both plaque number and diameter were increased in SOD2 KO cells (Figure 4B). At higher MOIs (1 and 5), fluorescent plaques in SOD2 KO cells continued to expand, often merging into larger structures, which precluded accurate diameter measurement but further underscored the enhanced intercellular transmission. These observations were corroborated by crystal violet plaque assays. At high MOI, extensive cytopathic effect precluded accurate plaque counting in SOD2 KO cells, but enhanced viral spread was evident (Figure 4C). At low MOI, SOD2 KO cells again produced significantly more and larger plaques than sgScram controls (Figure 4C,D). Collectively, these findings demonstrate that SOD2 deficiency promotes viral transmission and plaque expansion, whereas SOD2 restricts viral spread and cytopathic effect.

### 3.5. Ectopic Expression of SOD2 Restricts VACV Infection and Spread

**Figure 5:**
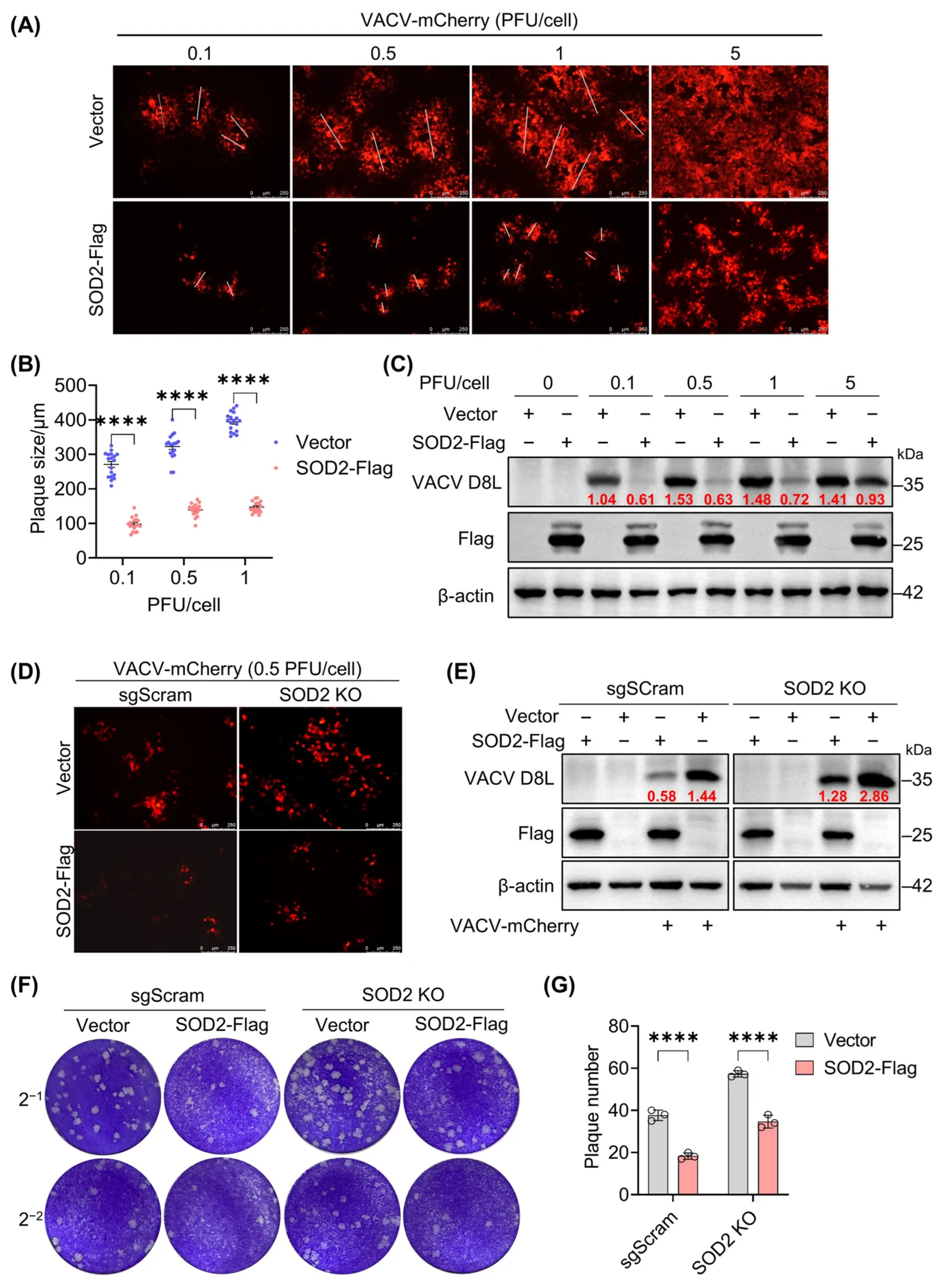
SOD2 overexpression limits viral infection and blocks viral spread. (**A**) Representative fluorescence images showing VACV infection in SOD2-overexpressing 293T cells and vector controls. Cells were transfected with SOD2-Flag or a control plasmid, infected with VACV-mCherry at the indicated MOIs, and imaged at 24 hpi. (**B**) Quantitative analysis of plaque sizes in panel (**A**); diameters were measured and statistically evaluated. Data represent mean ± SEM (n = 18 independent experiments). (**C**) Western blotting analysis of viral D8L protein expression in VACV-mCherry-infected SOD2-overexpressing 293T or vector control cells. Protein levels were normalized to β-actin and are presented relative to control. (**D**) Representative fluorescence images showing VACV-mCherry infection in SOD2-Flag-transfected A549 SOD2 KO and sgScram cells. Cells were transfected with SOD2-Flag or vector plasmid for 24 h, then infected with VACV-mCherry (MOI = 0.5 PFU/cell), and imaged at 24 hpi to evaluate viral infectivity. (**E**) Western blotting analysis of viral D8L protein expression in VACV-mCherry-infected SOD2-KO A549 or sgScram cells. Protein levels were normalized to β-actin and are presented relative to control. (**F**) Plaque assay of extracellular viral titers from SOD2-deficient or SOD2-reconstituted cells. A549 SOD2 KO and sgScram cells were transfected with SOD2-Flag or control plasmid for 24 h, infected with VACV at indicated MOIs. Supernatants collected at 24 hpi were filtered (0.45 μm) and analyzed by plaque assay. (**G**) Quantitative analysis of viral plaques from panel (**F**). Data represent mean ± SEM (n = 3 independent experiments except for the measurement of plaque size); **** *p* < 0.0001.

The effect of exogenous SOD2 on VACV infection was next assessed using a SOD2-encoding eukaryotic expression plasmid, with a C-terminal Flag tag incorporated to differentiate exogenous from endogenous SOD2 expression. 293T cells transfected with either SOD2-encoding or vector plasmids were challenged with VACV-mCherry at various MOIs. At 24 hpi, viral infectivity was evaluated by fluorescence plaque assays and viral protein expression analysis. SOD2 overexpression resulted in a significant reduction in fluorescent plaque numbers relative to vector controls (Figure 5A). At low MOIs, plaque diameters were markedly smaller in SOD2-expressing cells (Figure 5B), suggesting restricted intercellular viral spread. Concurrently, the expression of the VACV D8L protein was substantially diminished upon SOD2 overexpression (Figure 5C). Together, these results indicate that SOD2 potently inhibits VACV infection and replication. Functional rescue experiments in SOD2 KO cells further confirmed these findings. Fluorescent imaging analysis showed that the rescue of SOD2 expression in its KO cells significantly enhanced cellular resistance to VACV infection, as evidenced by a reduction in the number of infected cells (Figure 5D) and decreased intracellular viral D8L protein load (Figure 5E). Quantification of extracellular virus by titration assays revealed that SOD2 deficiency led to a substantial increase in the yield of extracellular mature virus (Figure 5F,G), which correlated with enhanced viral replication. Conversely, exogenous SOD2 expression in both sgScram and KO cells reduced extracellular mature virus production (Figure 5F,G), indicating that restoration of SOD2 function suppresses the release of mature viral particles. Collectively, the above findings demonstrate that SOD2 exerts the negative effect on vaccinia virus by suppressing viral replication and spread.

### 3.6. No Significant Correlation Between SOD2 Antiviral Activity and Its Dependence on Mitochondrial Localization or Manganese Binding

**Figure 6:**
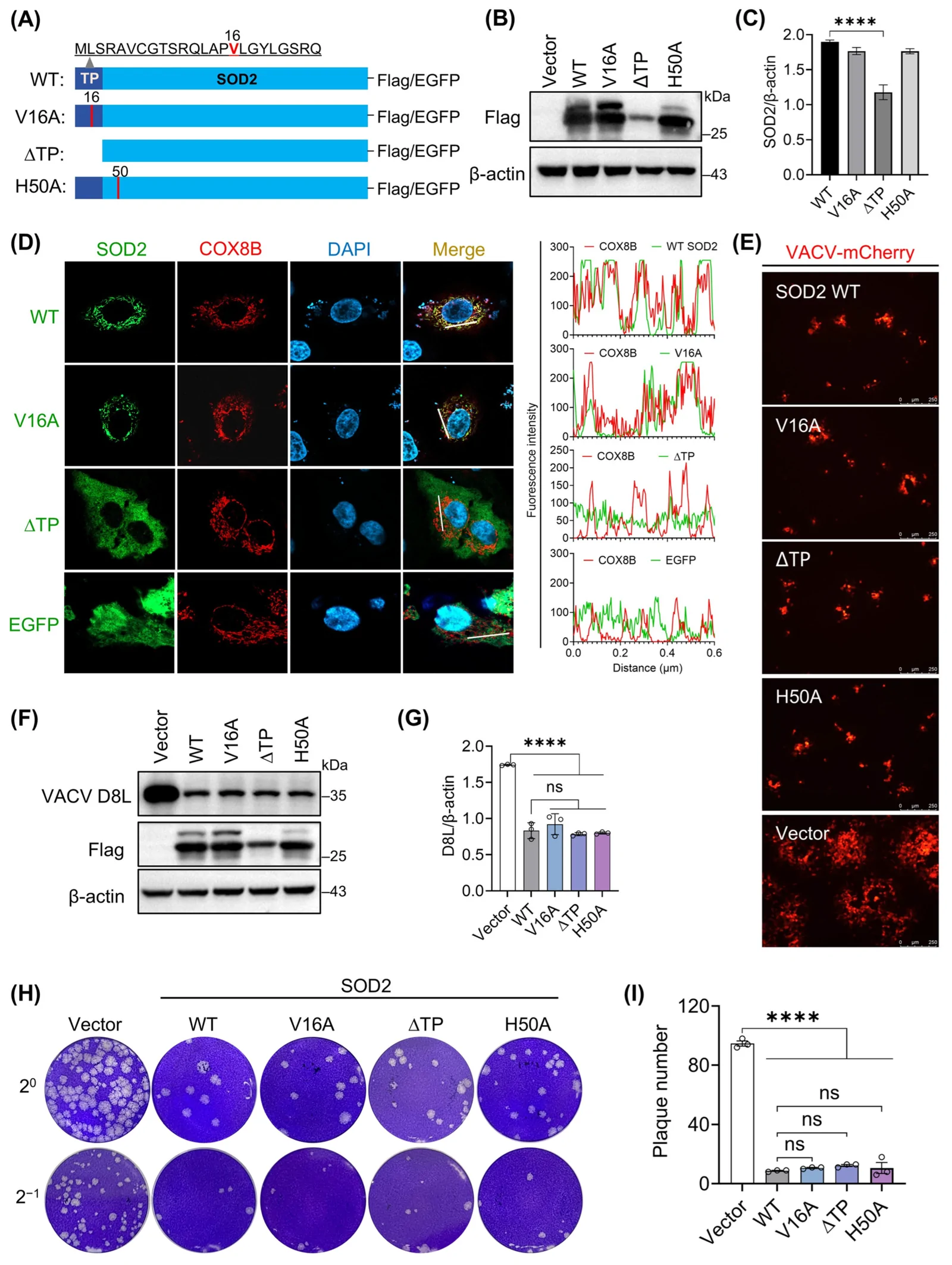
Neither mitochondrial localization nor manganese binding is required for the antiviral activity of SOD2. (**A**) Schematic representation of SOD2 mutants constructed based on the SOD2-Flag via the point mutation technology or truncation, including SOD2 V16A, ∆TP, and H50A. (**B**) Expression validation of SOD2 wild-type (WT) and mutants or truncation by Western blotting. (**C**) Quantitative analysis of expression levels of WT SOD2 and its mutants from panel (**B**). Protein levels were normalized to β-actin. (**D**) Subcellular localization of EGFP-tagged SOD2-WT and mutants in A549 cells. (**E**) Fluorescence imaging of VACV-mCherry infection in cells expressing wild-type or mutant SOD2. 293T cells expressing wild-type SOD2 or its mutant variants were infected with VACV-mCherry (0.5 PFU/cell). Viral infection was monitored by fluorescence microscopy at 24 hpi. (**F**) Western blotting analysis of viral D8L expression in virus-infected cells expressing wild-type or mutant SOD2. Cells expressing wild-type or mutant SOD2 were infected with VACV-mCherry (0.5 PFU/cell). At 24 hpi, cells and supernatants were harvested separately. Cell lysates were subjected to detect viral D8L protein expression. (**G**) Quantification of viral D8L protein levels from panel (**F**). Protein levels were normalized to β-actin. (**H**) Plaque assay analysis of viral titers in supernatants from infected cells expressing wild-type or mutant SOD2. Supernatants were collected from the experiment presented in panel (**E**). (**I**) Quantitative analysis of viral plaques. All data represent mean ± SEM (n = 3 independent experiments); **** *p* < 0.0001, ns indicates not significant.

SOD2 is a mitochondrial-localized manganese-binding protein. To determine whether its antiviral activity depends on its subcellular localization or manganese-binding capacity, we generated a series of SOD2 mutants and truncation variants, with a C-terminal Flag or enhanced green fluorescent protein (EGFP) tag for functional and localization studies (Figure 6A). The N-terminal transit peptide (TP) directs SOD2 to the mitochondrial matrix, where its cleavage yields the mature protein. The V16A mutation affects the secondary structure of the SOD2 mitochondrial targeting sequence (MTS) and has been reported to impair mitochondrial complex IV function in its carrier state [27,28]. Histidine 50 (H50), a core manganese-binding residue, abolishes enzymatic activity upon mutation (H50A) [29,30]. Western blot analysis revealed that mutants V16A and H50A exhibited expression levels comparable to wild-type (WT) SOD2, whereas the ΔTP mutant showed significantly reduced expression (Figure 6B,C), demonstrating that the TP is required for robust SOD2 expression in normal physiological states. Subcellular localization analysis revealed that WT SOD2 and its mutant V16A colocalized with mitochondrial marker COX8B, while the ΔTP mutant did not (Figure 6D), indicating that V16A mutation preserves mitochondrial targeting but loss of the TP abolishes mitochondrial targeting of SOD2, as previously reported [30].

Next, to evaluate whether mutations affect the antiviral activity of SOD2, cells expressing wild-type SOD2 and the SOD2 mutants (V16A, ΔTP, and H50A) were infected with VACV-mCherry at an MOI of 0.5, and viral infection was assessed by multiple parameters. Fluorescence imaging showed that all SOD2 variants markedly reduced the number of infected cells compared to empty vector controls, with no significant differences among the wild-type and mutant groups (Figure 6E), suggesting that these mutations do not substantially impair the antiviral function of SOD2. To corroborate this finding, intracellular viral protein levels and secreted viral titers (plaque assay) were quantified. Both wild-type SOD2 and its mutants significantly reduced viral D8L protein accumulation (Figure 6F,G) and extracellular mature virus production (Figure 6H,I) relative to vector controls, with comparable efficacy across all SOD2-expressing groups. These results show that mutation or even complete deletion of the TP, as well as alteration of the manganese-binding site, does not impair the antiviral activity of SOD2, indicating that the antiviral effect of SOD2 does not significantly correlate with its mitochondrial localization or manganese binding dependence.

### 3.7. K68 and K122 of SOD2 Play Important Roles in Mediating Its Antiviral Effect

**Figure 7:**
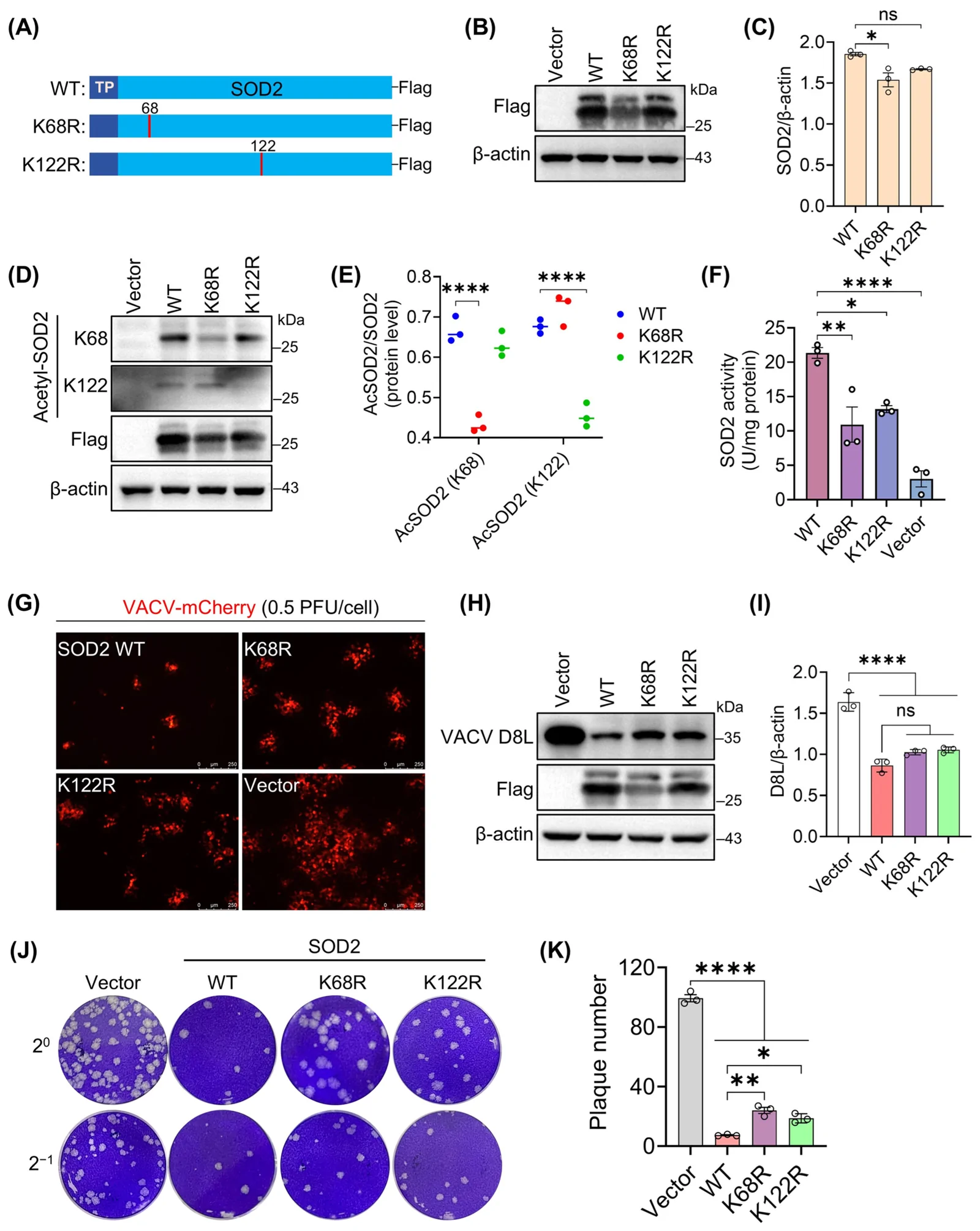
Lysine residues at positions 68 and 122 of SOD2 are critical for its antiviral activity. (**A**) Schematic representation of SOD2 acetylation site mutations, including K68 and K122, which were constructed based on the SOD2-Flag via the point mutation technology. (**B**) Expression validation of WT SOD2 or mutants K68R and K122R by Western blotting. (**C**) Quantitative analysis of expression levels of WT SOD2 and its mutants from panel (**B**). Protein levels were normalized to β-actin. (**D**) Acetylation status of K68R and K122R mutants detected using Acetyl-SOD2/MnSOD (K68) antibody and Acetyl-SOD2/MnSOD (K122) antibody, respectively. (**E**) Quantification of acetylation levels in cells expressing WT SOD2 or mutants (K68R or K122R) from panel (**D**). Protein levels were normalized to flag-tagged SOD2. (**F**) SOD2 activity assay in cells expressing WT SOD2 or mutants (K68R and K122R). (**G**) Fluorescence imaging of VACV-mCherry infection in cells expressing WT SOD2 or mutants (K68R or K122R). 293T cells expressing WT SOD2 or its mutants were infected with VACV-mCherry (0.5 PFU/cell), respectively. Viral infection was monitored by fluorescence microscopy at 24 hpi. (**H**) Western blotting analysis of viral D8L expression in virus-infected cells expressing WT SOD2 or mutants. Cells expressing WT SOD2 or mutants were infected with VACV-mCherry (0.5 PFU/cell). At 24 hpi, cells and supernatants were harvested separately. Cell lysates were subjected to detect viral D8L protein expression. (**I**) Quantification of viral D8L protein levels from panel (**H**). Protein levels were normalized to β-actin. (**J**) Plaque assay analysis of viral titers in supernatants from infected cells expressing wild-type or mutant SOD2. Supernatants were collected from the experiment presented in panel (**H**). (**K**) Quantitative analysis of viral plaques from panel (**J**). All data represent mean ± SEM (n = 3 independent experiments); * *p* < 0.05; ** *p* < 0.01; **** *p* < 0.0001; ns indicates not significant.

We further focused on the regulation of SOD2 activity by post-translational modifications, particularly lysine (K) acetylation at residues including K68 and K122 [31,32]. Acetylation at these sites has been shown to attenuate the antioxidant activity of SOD2, leading to elevated mitochondrial superoxide levels [33,34]. Next, to investigate whether acetylation at these sites modulates its antiviral activity, we generated K68R and K122R SOD2 mutants with a C-terminal Flag tag for functional studies (Figure 7A), in which K was replaced by arginine (R) to mimic a constitutive deacetylated status. Successful expression of both SOD2 mutants was confirmed by Western blotting (Figure 7B), with the K68R mutant showing notably reduced protein levels (Figure 7C), indicating a possible link between K68 and SOD2 stability. Acetylation-specific analysis revealed that both K68R and K122R mutants exhibited significantly lower acetylation than wild-type SOD2 (Figure 7D,E), consistent with their design as deacetylation-mimicking variants. Correlating with this, enzymatic activity assays demonstrated a significant loss of SOD2 function upon mutation of either K68 or K122 (Figure 7F), establishing the critical importance of acetylation at these residues for proper antioxidant activity.

To investigate whether K68R and K122R mutations alter the anti-poxviral function of SOD2, 293T cells expressing wild-type SOD2 or its mutants were infected with VACV (MOI = 0.5 PFU/cell) for 24 h, and viral replication was evaluated using complementary assays. Fluorescence imaging and viral protein analysis showed a slight, non-significant increase in infected cells and viral protein levels in mutant-expressing cells compared to wild-type controls (Figure 7G–I). Extracellular viral titration assays revealed that cells transfected with K68R or K122R mutants produced and released significantly more infectious viral particles, as evidenced by increased plaque formation, compared to wild-type SOD2-expressing cells (Figure 7J,K). These findings indicate that mutations at K68 and K122 significantly impair the capacity of SOD2 to suppress viral replication and extracellular mature virus production, suggesting that K68 and K122 are residues that contribute to SOD2-mediated restriction of viral replication and yield.

### 3.8. Local Pretreatment with SOD2 Significantly Suppressed Poxvirus Lesion Formation on Rabbit Skin

**Figure 8:**
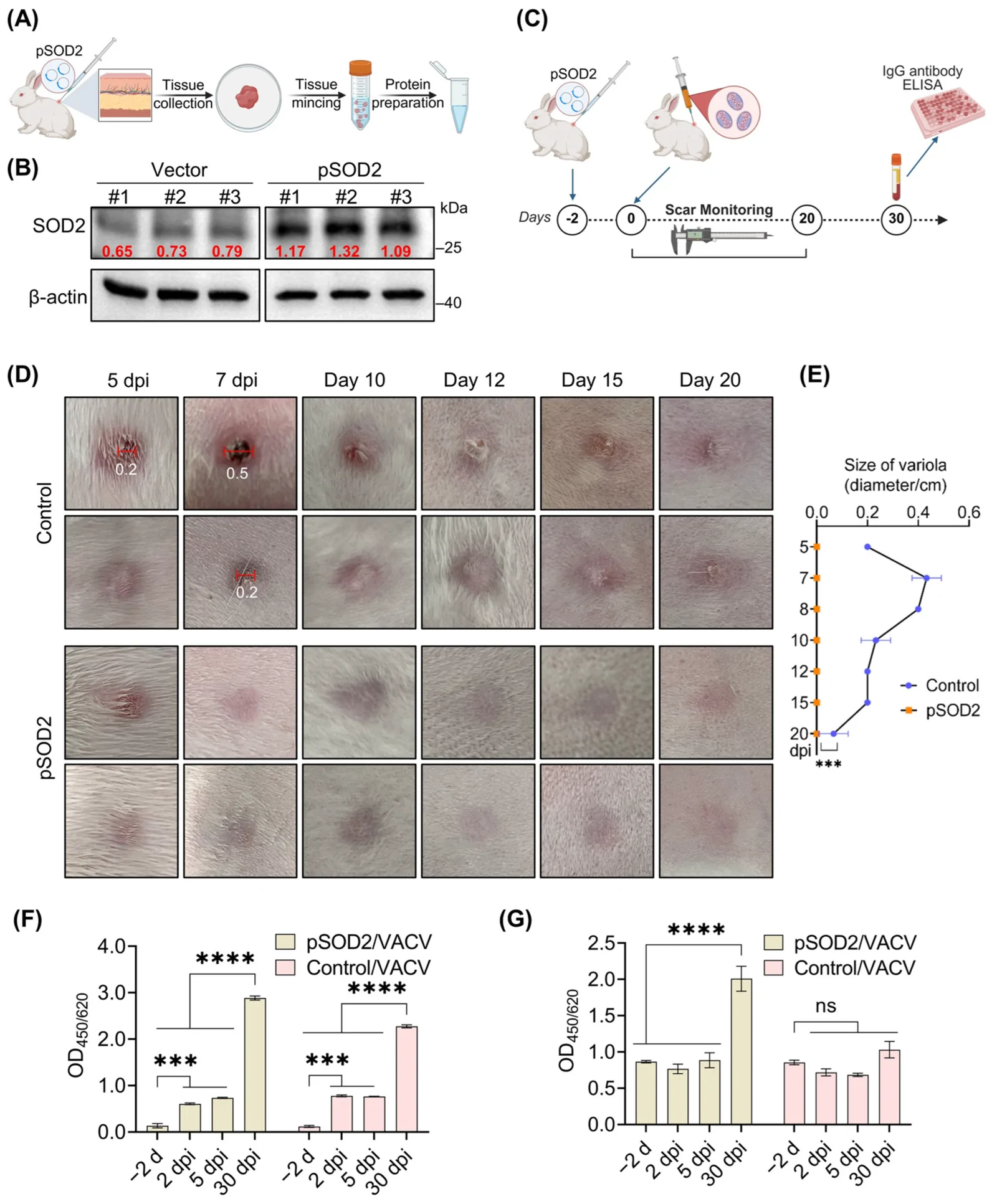
Local pretreatment with SOD2 significantly suppressed VACV lesion formation on rabbit skin. (**A**) Schematic workflow of intradermal injection of pSOD2 plasmid-liposome complexes in rabbits and analysis of tissue protein expression. (**B**) Detection of SOD2 expression in skin tissues after local injection of SOD2 plasmid-liposome complexes. (**C**) Schematic workflow of VACV challenge in rabbit skin preinjected with pSOD2-liposome complexes, with timelines for lesion monitoring and serological sampling. Intradermal injection of the liposome-plasmid complex (30 μg) was administered into the back of rabbits. 2 d later, VACV (1 × 10^6^ PFU, 100 μL) was injected intradermally into the same injection site; serum samples were harvested from the marginal ear vein of rabbits at 2, 5, and 30 days after infection. (**D**) Representative images of skin lesion formation in SOD2-treated and control rabbits. Comparison of acne spot formation on the backs of rabbits in the SOD2-treated group and the control group at different time points after challenge. (**E**) Changes in lesions over time. (**F**,**G**) Detection of VACV-specific (**E**) and human SOD2-specific (**F**) antibodies in rabbit serum samples. All data represent mean ± SEM (n = 3); *** *p* < 0.001; **** *p* < 0.0001; ns indicates not significant. (**A**,**C**) were created in BioRender. Shouwen Du. (2026). BioRender.com/3iv8lcm.

The above in vitro studies demonstrated that SOD2 significantly inhibits VACV infection and replication. To determine whether SOD2 exhibits comparable antiviral activity under physiological conditions, we subsequently established a rabbit model with localized SOD2 expression in the skin and performed VACV skin infection assays. Thus, we first established a model of localized SOD2 expression in rabbit skin. Liposome-SOD2 plasmid (pSOD2) complexes or control formulations prepared with Lipofectamine 3000 were injected intradermally into depilated dorsal skin (Figure 8A). At 48 h post-injection, skin biopsies were collected from injection sites, and protein extracts were analyzed for human SOD2 expression. Human SOD2 levels were markedly elevated at sites receiving pSOD2 compared to vector controls (Figure 8B), confirming successful local expression.

Subsequently, we challenged these sites with intradermal inoculation of VACV (1 × 10^6^ PFU) and monitored lesion progression over time (Figure 8C). Control rabbits developed characteristic vaccinia lesions, with prominent redness, swelling, ulceration, and scab formation by day 5–7, and visible tissue protrusions persisting until day 20 (Figure 8D). In striking contrast, SOD2-treated rabbits exhibited only mild erythema at day 5, with no ulceration or scab formation throughout the observation period. Wound healing was accelerated, with near-complete restoration of normal skin architecture by day 20 (Figure 8D,E), indicating that localized human SOD2 expression effectively attenuates VACV-induced skin damage. Additionally, the antibody profiling showed that poxvirus-specific antibodies appeared in serum by day 2 post-infection and peaked at day 30 (Figure 8F). Notably, significant levels of anti-human SOD2 antibodies were detected in sera from SOD2 plasmid-injected rabbits at day 30 post-infection (Figure 8G), providing further evidence for successful expression of the human protein in rabbit skin. Together, these findings suggest that topical SOD2 intervention confers protection against poxvirus-induced skin lesions in this rabbit model, underscoring its therapeutic potential.

## 4. Discussion

**Figure 9:**
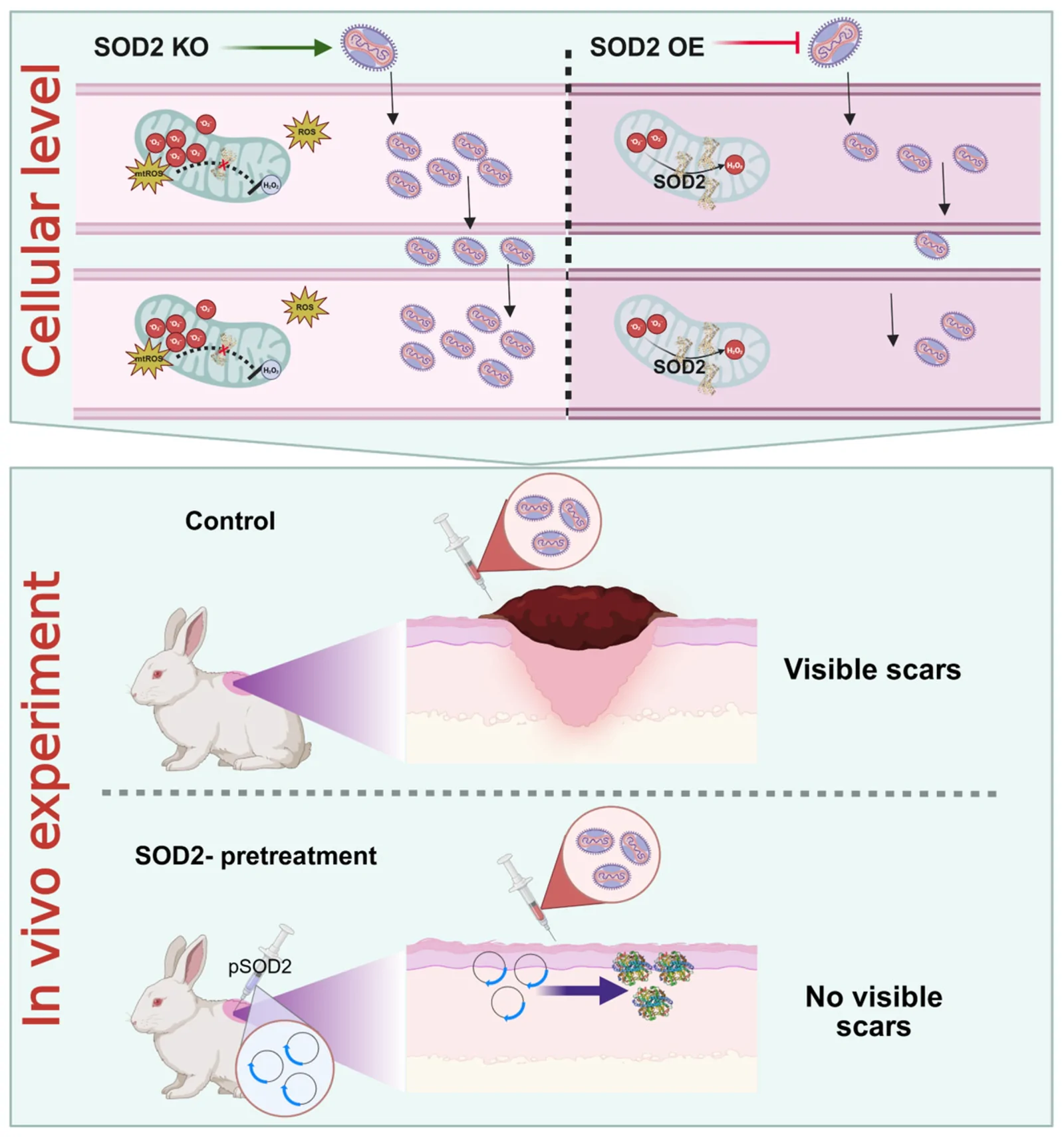
Schematic model illustrating the inhibitory effect of SOD2 on VACV replication, propagation, and pathogenicity, as demonstrated by in vitro cellular level and in vivo animal experiments. Loss- and gain-of-function approaches demonstrated that SOD2 negatively regulates VACV cell-to-cell transmission at the cellular level, establishing an inverse relationship between SOD2 expression and viral spread. Consistent with this, SOD2-pretreated rabbits showed no visible scarring after viral inoculation, in stark contrast to control animals that developed pronounced lesions. Created in BioRender. Shounwen Du. (2026). BioRender.com/8bxqzzn.

This study provides the first lines of evidence at both cellular and animal levels suggesting that SOD2 can act as a host factor that limits a poxvirus infection and pathogenicity under the experimental conditions tested (Figure 9). These findings reveal that, in addition to its canonical role in maintaining redox homeostasis, SOD2 possesses antiviral activity that suppresses VACV replication and propagation. Notably, this antiviral function is not strictly dependent on its mitochondrial localization or manganese-binding capacity. However, this does not imply that the anti-VACV activity of SOD2, or viral replication and proliferation, are unrelated to intracellular redox homeostasis; quite the contrary, a close interplay exists between these processes. Furthermore, the sustained global threat of mpox [35,36] has amplified public health concerns and redirected focus to orthopoxviruses, including the eradicated smallpox virus [37]. This reality highlights the pressing requirement to elucidate poxvirus-host interactions for the development of effective therapeutic interventions.

Oxidative stress regulation is one of the core components of orthopoxvirus-host interactions. In this context, we investigated whether the host antioxidant enzyme SOD2 or the viral-encoded Cu-Zn superoxide dismutase-like protein (e.g., VACV Tiantan strain TA56R) plays a dominant role in redox modulation during VACV infection. Our results demonstrate that host SOD2 serves as the principal ROS scavenger, whereas the viral superoxide dismutase-like protein, despite its sequence homology to SOD, lacks intrinsic enzymatic activity and fails to reduce mitochondrial ROS levels. These findings are consistent with previous reports [25] and collectively indicate that the viral homolog cannot compensate for the loss of host SOD2 function, underscoring the non-redundant and essential role of endogenous SOD2 in host defense against VACV-induced oxidative stress.

This study provided an exploratory elucidation of the correlation between host SOD2 and VACV infection and revealed a distinct phenomenon of poxvirus-mediated modulation of reactive oxygen species (ROS) that differs from some previous reports [13]. Notably, this heterogeneity was also observed among cells with varying infection levels, suggesting that it may be associated with differential activation of the endogenous antioxidant defense system in infected cells. Interestingly, VACV infection selectively induced SOD2 upregulation without significantly affecting SOD1 expression. This differential regulation led us to focus on SOD2 as a potential mediator in viral infection and associated oxidative stress responses. To date, the only known enzyme to successfully remediate O_2_^•−^ radicals within mitochondria is SOD2 (MnSOD), which dismutes two O_2_^•−^ radicals into molecular oxygen (O_2_) and hydrogen peroxide (H_2_O_2_) [38]. Its protective role against oxidative stress is critical for mitochondrial function, cellular health and survival, as evidenced by studies that show complete knockout of the SOD2 gene results in early mortality in mice due to reduced activity of several mitochondrial proteins [39,40]. In this study, we established and validated SOD2 knockout cell lines, confirming complete loss of SOD2 protein without impacting cell viability. Importantly, SOD2 deficiency disrupted redox homeostasis, elevating basal and VACV-induced ROS levels. This oxidative shift suggests that SOD2 may function as a key modulator of the cellular environment during viral infection, raising the question of whether its absence directly enhances viral pathogenicity through redox-dependent mechanisms.

Accumulating evidence establishes a causal link between redox status and antiviral innate immunity. Specifically, SOD2 deficiency leads to mitochondrial ROS accumulation, which impairs RIG-I-like receptor (RLR)-mediated type I interferon production and thereby enhances viral replication [41]. Concurrently, mitochondrial ROS levels directly modulate MAVS signaling and subsequent NF-κB/IRF3 activation, which are essential for restricting viral replication [42]. Thus, the enhanced VACV replication in SOD2-KO cells observed in our study is likely attributable to ROS-mediated suppression of innate immunity. This causal framework does not require additional experimentation, as it is firmly supported by published literature. The role of SOD2 in antiviral defense is further corroborated across diverse experimental systems: engagement of TLR2, TLR3, TLR4, and TLR7/8 all upregulate SOD2 expression [43], with TLR3 selectively inducing SOD2 as the dominant antioxidant response in astrocytes [44]. In vivo evidence underscores its physiological relevance, as T cell-specific SOD2 knockout increases susceptibility to influenza A virus [45]. Conversely, viruses have evolved countermeasures: HIV-1 Tat represses SOD2 transcription [46] and respiratory syncytial virus NS1 suppresses SOD2 induction further implicating SOD2 as a key target of viral immune evasion [47]. In our experiment, SOD2 knockout resulted in increased ROS production and enhanced VACV replication. This result suggests that ROS-mediated innate immune suppression may help promote viral growth. Notably, however, our mutant analyses reveal that SOD2 restricts VACV through mechanisms not strictly dependent on its enzymatic activity, pointing to a mode of action that extends beyond canonical ROS scavenging and appears distinct from the paradigm established for RNA viruses.

Using the SOD2 knockout system, we observed that SOD2 deficiency significantly enhanced VACV infection, as evidenced by increased viral protein expression and augmented plaque formation compared to control cells. Quantitative analysis revealed that at a low multiplicity of infection (MOI), SOD2 KO cells exhibited both increased plaque numbers and enlarged plaque diameters. Conversely, at high MOI, SOD2 KO cells formed substantially larger but fewer plaques, consistent with accelerated viral transmission leading to plaque fusion. These findings demonstrate that SOD2 loss promotes viral infectivity and spread, while SOD2 overexpression conversely suppresses VACV propagation. Collectively, our results establish SOD2 as a critical host factor restricting poxvirus replication and dissemination in vitro.

Mechanistically, we initially focused on its key functional domains, generating a series of mutants via point mutations or truncations targeting residues critical for its canonical enzymatic activity, including the mitochondrial localization signal and manganese-binding sites. Previous studies have established that mitochondrial targeting and manganese coordination are essential for the classical antioxidant function of SOD2 [48,49]. Unexpectedly, however, neither disruption of mitochondrial localization nor mutation of the manganese-binding residues significantly altered its antiviral activity, suggesting that the antiviral effect of SOD2 operates independently of its conventional enzymatic function. In parallel, we assessed the contribution of other functionally important residues (specifically K68 and K122) to SOD2-mediated viral restriction. These sites are known to undergo acylation/deacylation modifications and serve as key regulatory switches for SOD2′s antioxidant defense capacity [31,50]. By generating K68R and K122R point mutants to mimic the deacetylated state, we confirmed that these mutations indeed reduced both acetylation levels and enzymatic activity of SOD2, as expected. Notably, although both mutants retained substantial viral suppression capacity, their antiviral activity was significantly attenuated compared to wild-type SOD2. This observation indicates that post-translational modifications at specific lysine residues contribute to the antiviral function of SOD2. The retention of antiviral activity in mitochondrial localization and manganese-binding mutants suggests that SOD2 restricts VACV through mechanisms decoupled from its canonical enzymatic function. This is consistent with emerging evidence that SOD2 can act as a signaling scaffold or redox sensor independent of superoxide dismutation. Conversely, the attenuated but not abolished antiviral activity of K68R and K122R mutants indicates that acetylation at these residues fine-tunes but does not exclusively govern the antiviral phenotype. The discrepancy between our observed enzymatic reduction in these mutants and prior reports of activation may reflect cell type–specific folding constraints or virus-induced alterations in the mitochondrial microenvironment. Collectively, these data support a model in which SOD2-mediated restriction of poxvirus replication relies on a combination of catalytic and non-catalytic functions, the relative contributions of which remain to be fully dissected. However, the precise molecular events downstream of SOD2 are not fully resolved. Whether SOD2 restricts viral replication by directly engaging viral or host proteins, modulating specific metabolic pathways, or regulating redox-sensitive transcription factors remains open. In addition, our mechanistic experiments were performed in a single immortalized cell line using the laboratory-adapted VACV Tiantan strain; whether these findings extend to primary cells, other tissue types, or other orthopoxviruses such as MPXV awaits further investigation.

In addition, several considerations regarding the use of human SOD2 in rabbits merit discussion. First, pairwise BLAST (BLASTP 2.17.0+) alignment of human (P04179) and rabbit (P41982) SOD2 protein sequences revealed 92.04% amino acid identity over 91% of the query length (E-value = 3 × 10^−146^), indicating a high degree of evolutionary conservation (Figure S3). Moreover, all functional residues examined are fully conserved, supporting functional equivalence. Second, although xenogeneic proteins can elicit immune responses, we detected only low titers of anti-human SOD2 antibodies by day 30 post-infection, and the protective effect was already evident at day 5–7, arguing against a major contribution of immune-mediated artifacts. Nevertheless, we cannot formally exclude the possibility that minor sequence differences influence protein–protein interactions or that protein overexpression itself may have non-specific effects. Although our study provides clear evidence that SOD2 restricts VACV replication in vitro and attenuates poxvirus pathogenicity in a rabbit model, these findings were generated exclusively in the context of a single orthopoxvirus. It is important to note that SOD2 has been reported to facilitate antiviral innate immunity against RNA viruses by preserving mitochondrial redox balance and supporting RIG-I-like receptor signaling [41]; however, DNA viruses such as poxviruses employ distinct replication strategies and immune evasion mechanisms, including active modulation of host oxidative stress responses. Consequently, the antiviral function of SOD2 observed here may not be universally applicable across all viral families, and its efficacy against phylogenetically distant pathogens remains speculative. Systematic investigation across diverse viral families will be necessary to determine whether SOD2 represents a general restriction factor or a context-specific modulator of virus–host interactions.

This study has several limitations. First, the molecular mechanism of SOD2-mediated restriction remains incompletely defined. Second, all experiments were conducted in vitro or in a cutaneous rabbit model, leaving systemic pathogenesis and immune dynamics unexplored. Third, the antiviral function of SOD2 was assessed only against VACV. Future work should integrate AP-MS and metabolomics to dissect mechanism, extend validation to other orthopoxviruses and RNA viruses, and employ genetic models (e.g., SOD2 conditional knockout rabbits) to establish physiological relevance. From a translational perspective, VACV and its highly attenuated derivatives (MVA, NYVAC) have been widely used as vaccine vectors in humans and other mammals [51]. The safety profiles of these modified attenuated strains in clinical trials support their continued development. Understanding how host factors such as SOD2 restrict poxvirus replication may inform strategies to enhance vaccine vector immunogenicity or develop host-directed antiviral therapeutics against orthopoxvirus infections, including mpox.

## 5. Conclusions

In summary, our study revealed that SOD2 acts as a host restriction factor against vaccinia virus infection through mechanisms that are not entirely identical to its classical antioxidant activity. By establishing a rabbit model with cutaneous SOD2 expression via DNA-based delivery, we provide in vivo evidence that exogenous SOD2 markedly attenuates the poxvirus pathogenicity. This work uncovers a previously overlooked antiviral function of SOD2, which underscores its potential utility in the development of biologic therapeutics against orthopoxvirus infections.

## 6. Patents

A patent application related to this work has been filed in China (Application No. 202511529334.2, filed 9 January 2026). The authors declare no competing interests.

## Figures and Tables

**Table 1:** Primer Design and Synthesis (NCBI RefSeq: NM_000636.4).

Primers	Sequence (5′-3′)
sgRNA-F	CACCACGTGAACAACCTGAACGTC
sgRNA-R	AAACGACGTTCAGGTTGTTCACGT
SOD2-F	ATAGATCTGATATCGGTACCATGTTGAGCCGGGCAGTG
SOD2-R	AGTCAGCCCGGGATCCCTTTTTGCAAGCCATGTATCTT
ΔTP-F	ATAGATCTGATATCGGTACCATGAAGCACAGCCTCCCC
ΔTP-R	AGTCAGCCCGGGATCCCTTTTTGCAAGCCATGTATCTT
V16A-F	AGCTGGCTCCGGCGTTGGGGTATCTGGGCTCCA
V16A-R	ACCCCAAAACGCGAGCCAGCTGCCTGCTGGTGC
H50A-F	ATCATGCAGCTGGCGCACAGCAAGCACCACGCG
H50A-R	TGCGCCAGCTGCATGATCTGCGCGTTGATGTG
K68R-F	GAGGAGCGCTACCAGGAGGCGTTGGCCAAGGG
K68R-R	TCCTGGTAGCGCTCCTCGGTGACGTTCAGGTT
K122R-F	CCATCCGCCGTGACTTTGGTTCCTTTGACAAG
K122R-R	AAAGTCACGGCGGATGGCTTCCAGCAACTCCC

## Data Availability

Data is contained within the article or supplementary material.

## Supplementary Materials

The following supporting information can be downloaded at: https://www.mdpi.com/article/10.3390/antiox15081019/s1, Figure S1: Construction of SOD2 knockout cell lines; Figure S2: Sequence alignment of the SOD-like protein encoded by the VACV A45R gene (TA56R) with the SOD2 gene sequence; Figure S3: Pairwise alignment of human (P04179) and rabbit (P41982) SOD2 protein sequences.

## Abbreviations

The following abbreviations are used in this manuscript:

SOD1	Superoxide dismutase 1
SOD2	Superoxide dismutase 2
VACV	Vaccinia virus
MPXV	Monkeypox virus
VARV	Variola virus
CPXV	Cowpox virus
ROS	Reactive oxygen species
ELISA	Enzyme-Linked Immunosorbent Assays
sgRNA	Single guide RNA
MOI	Multiplicity of infection
KO	Knock out
WT	Wild-type
