# Supplementary material for: The Important Role of Superoxide Dismutase 2 in Controlling Poxvirus Proliferation and Pathogenicity
Source: Antioxidants (Basel). 2026 Aug 15;15(8):1019. doi: 10.3390/antiox15081019 (PMC13509771; doi:10.3390/antiox15081019)
Supplement: Supplementary file 1 [file antioxidants-15-01019-s001.zip › antioxidants-4451937-supplementary.pdf]

Supplemental materials:

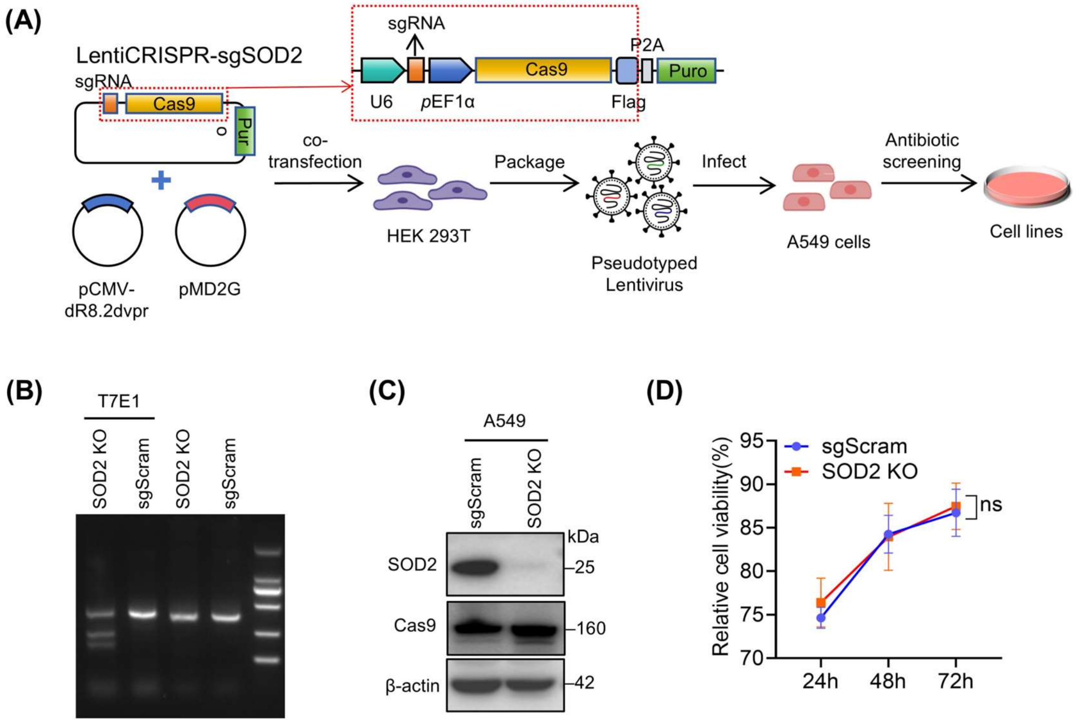

Supplemental Figure S1. Construction of SOD2 knockout cell lines. (A) Schematic diagram of the SOD2 knockout cell line construction process. (B) Genotype identification using T7 Endonuclease I assay in A549-SOD2 KO cells. (C) SOD2 protein expression analysis in A549-SOD2 KO and control cells. (D) Cell viability assessment by CCK-8 assay at 24, 48, and 72 hours.

```
SOD2   1    MLSRAVCGTSRQLAPVLGYLGSRQKHSLPDLPYDYGALEPHINAQIMQLH
TA56R  1    M---AVCIIDHD--NIRGVIYFEPVHG-----------KDKVLGSVIGLK

SOD2   51   HSKHHAAYVNNLNVTEEKYQEALAKGDVTAQIALQPALKFNGGGHI-NHS
TA56R  35   SGTY--------SLIIHRY------GDIS-----------QGCDSIGSPE

SOD2   100  IFWTNLSPNGGGEPKGELLEAIKRDFGSFDKFKEKLTAASVGVQGSGWGW
TA56R   60  IFIGNIFVN----RYGVAYVYLDTDVNIFTIIGKALSISK----------

SOD2   150  LGFNKERGHLQIAACPNQDPLQGTTGLIPLLGIDVWEHAYYLQYKNVRPD
TA56R   95  ---NDQR-----LAC-------------EVIGIS------YINEK-----

SOD2   200  YLKAIWNVINWENVTERYMACKK
TA56R  113  -------IIHFLTINENGV----
```

Supplemental Figure S2. Sequence alignment of the Cu-Zn superoxide dismutase-like protein encoded by the TA56R gene of VACV Tiantan strain with the human SOD2 gene sequence.

```
SOD2-HUMAN    MLSRAVCGTSRQLAPVLGYLGSRQKHSLPDLPYDYGALEPHINAQIMQLHHSKHHAAYVN   60
SOD2-RABBIT   -------------------HGRGMKHSLPDLPYDYGALEPHINAQIMELHHSKHHAAYVN   41

SOD2-HUMAN    NLNVTEEKYQEALAKGDVTAQIALQPALKFNGGGHINHSIFWTNLSPNGGGEPKGELLEA  120
SOD2-RABBIT   NLNATEEKYREALARGDVTAHVALQPALKFKGGGHINHTIFWTNLSPNGGGEPKGELLEA  101

SOD2-HUMAN    IKRDFGSFDKFKEKLTAASVGVQGSGWGWLGFNKERGHLQIAACPNQDPLQGTTGLIPLL  180
SOD2-RABBIT   IKRDFGSFDKFKERLTAVSVGVQGSGWGWLGFNKEQGHLQIAACANQDPLQGTTGLIPLL  161

SOD2-HUMAN    GIDVWEHAYYLQYKNVRPDYLKAIWNVINWENVTERYMACKK                    222
SOD2-RABBIT   GIDVWEHAYYLQYKNVRPDYLKAIWNVITWENVTERYMACK-                    202


Percent Coverage:  91%
Evalue:            3e-146
Percent Identity:  92.04  %
```

Supplemental Figure S3. Pairwise alignment of human (P04179) and rabbit (P41982) SOD2 protein sequences. The alignment shows a high degree of evolutionary conservation, with 92.04% amino acid identity over 91% of the query sequence (E-value = 3e-146).
